# Teclistamab interference with anti-BCMA chimeric antigen receptor T-cell detection by flow cytometry: duration and clinical implications

**DOI:** 10.1038/s41375-026-02961-y

**Published:** 2026-04-08

**Authors:** Jonas Schadt, Malte von Bonin, Uta Oelschlägel, Raphael Teipel, Katharina Epp, Felix Carl Saalfeld, Freya Schulze, Sandra Heuschkel, Martin Bornhäuser, Maximilian Alexander Röhnert

**Affiliations:** 1 Department of Medicine I, Medical Faculty and University Hospital Carl Gustav Carus, TUD Dresden University of Technology, Dresden, Germany; 2 Hospital pharmacy, Medical Faculty and University Hospital Carl Gustav Carus, TUD Dresden University of Technology, Dresden, Germany; 3 National Center of Tumor Diseases, Dresden, Germany

**Keywords:** Cancer immunotherapy, Immunotherapy, Myeloma

## Introduction

Teclistamab is a bi-specific antibody targeting CD3 and B-cell maturation antigen (BCMA), used in relapsed or refractory multiple myeloma (MM) beyond first-line therapy. Even though it achieves response rates of up to 63% [1], relapses are common with a median progression-free survival of less than 1 year. Therefore, most of these patients require further lines of therapy. One option is BCMA-directed chimeric antigen receptor (CAR) T-cell therapy. In Europe, two commercial products are currently approved for this purpose (Abecma® and Carvykti®). Additionally, further BCMA-CARs are in development, so the variety of options may increase in the coming years [2].

As side effects and efficacy of CAR T-cell therapy may correlate with CAR T-cell expansion and persistence, measurement of circulating CAR T-cells in peripheral blood is widely established and recommended [3–6]. Besides polymerase chain reation (PCR) analyses, multicolor flow cytometry (MFC) has become a commonly used method for monitoring circulating CAR T-cells in routine diagnostics, as it is readily available, affordable, and fast [7]. For detection by MFC, various target structures can be used depending on the specific BCMA CAR T-cell product employed. However, the most commonly used approach is labeled human BCMA protein [8], as it not only works universally for all anti-BCMA CARs but also confirms functional antigen recognition.

As already described by Glatte et al. (2023) Teclistamab may cause false positive results in this MFC CAR T-cell detection assays by binding on circulating CD3^+^ T-cells but not BCMA-antigen and therefore presenting a free anti-BMCA arm [9]. Yet it’s unknown how long these false positive results will last after the last Teclistamab administration. Glatte et al. report wrong positive results in more than 95% of T-cells up to 8 days after the last Teclistamab administration. Taking the half-life of Teclistamab of 27 days into account [10], a much longer impairment of anti-BCMA CAR detection assays can be assumed. Therefore, we aimed to quantify false positive results in BCMA-CAR detection over time after Teclistamab therapy ceased.

## Methods

This study included MM patients before their first anti-BCMA CAR therapy who had received their most recent dose of Teclistamab within 14 days to 1 year prior to inclusion. Peripheral blood samples were collected in EDTA tubes between April 2024 and March 2025 as part of routine care blood counts and were subsequently utilized for MFC analyses within the study. Additional clinical data, like cumulative dose of Teclistamab, body weight, and blood counts, were retrieved from electronic treatment records.

Sample preparation followed an established protocol with 2 tubes according to the fluorescence minus one (FMO) concept: Around 1 000 000 white blood cells each were transferred into two tubes labeled (1) *FMO control* and (2) *BCMA-CAR detection*. Subsequently, 1 µL of BCMA-CAR detection reagent (see Supplemental Table S1 for details) was added to tube number 2. This tube was incubated for 15 min at room temperature (RT) in the dark, and cells were washed once with phosphate-buffered saline (Gibco™ PBS, pH 7,4, ThermoFischer, Waltham, MA, USA, Cat# 10010031). The remaining antibodies (CD3, Biotin, and CD45) were then added to both tubes, and samples were incubated again for 15 min at RT in the dark. Next, 2 mL of BD Pharm Lyse™ (1:10 dilution with distilled water; BD Biosciences, San Jose, CA, USA, Cat# 555899) was added to each tube, and the samples were incubated for an additional 10 min at RT in the dark. Cells were then washed twice with PBS. All stained samples were stored at 4 °C until flow cytometric analysis, which took place on the same day as blood drawing.

**Fig. 1:**
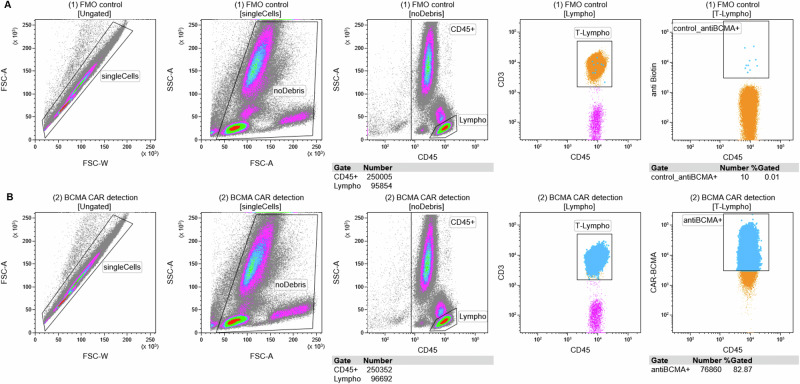
Gating strategy. **A** Tube 1 is used as a fluorescence minus one control (lacking BCMA^a^-CAR^b^ detection reagent) and gates are set accordingly. Notably, even though no BCMA-CAR detection reagent was added, some events may still be found within the BCMA^+^ T-lymphocyte gate in tube 1 (upper right panel). **B** Gates from Tube 1 are identically applied to Tube 2, which includes the BCMA-CAR detection reagent. The example depicted belongs to a patient 27 days after the last Teclistamab injection. The majority of T-lymphocytes are still anti-BCMA^+^ (false positive). ^a^BCMA: B-cell maturation antigen. ^b^CAR: chimeric antigen receptor.

All samples were measured centrally using a BD FACSCanto™ II flow cytometer and BD FACSDiva™ software (both Becton Dickinson, Franklin Lakes, NJ, USA). For analysis, Kaluza Analysis 2.1 software (Beckman Coulter, Brea, CA, USA) was used. Tube 1, as a negative control, guided the individual gates, which were then identically applied to Tube 2 (see Fig.1). After exclusion of debris and doublets by forward and sideward scatter, CD45^+^ Leukocytes were identified. Subsequently, all lymphocytes (CD45^+^SSC^low^), T-lymphocytes (CD45^+^CD3^+^SSC^low^), as well as anti-BCMA^+^ T-lymphocytes were identified. To ensure comparability between individual patients, the threshold between anti-BCMA-positive and -negative was set at a fixed fluorescence intensity of 3 000 following review of all negative controls. Even though no BCMA-CAR detection reagent was added, some events may still be found within the BCMA^+^ T-lymphocyte gate in tube 1. The percentage of BCMA^+^CD3^+^ cells among all CD3^+^ cells within this negative control was later subtracted from the percentage of BCMA^+^CD3^+^ cells in the second tube, giving the true percentage of BCMA^+^ T-cells. To ensure reliability, the population of BCMA^+^ T-Cells has to encompass at least 20 events.

To validate the assay, we performed negative controls with samples from healthy volunteers and MM patients who had never received BCMA-directed therapy (Supplemental Fig. S3). Positive controls were performed with MM patients recently treated with a BCMA-directed CAR-therapy and who had never been exposed to Teclistamab (Supplemental Fig. S4).

## Results

We were able to collect 13 samples from 11 different patients (one patient contributed 3 consecutive samples) after cessation of Teclistamab therapy, ranging from day 14 to day 357 (median 55 days). In median, patients received 18 Teclistamab injections, including step-up doses before sample collection (interquartile range 8 to 24 injections). The dosage of Teclistamab ranged from 1.5 mg/kg to 3 mg/kg body weight, administered in weekly to 4-weekly schedules. Details on patient characteristics and previous treatment scheme can be found in Supplemental Table S2.

In the 9 samples measured up to day 74 after the last Teclistamab application, we were able to identify significant amounts of anti-BCMA^+^ T-cells. Hereby, the detected proportion of anti-BCMA^+^ T-cells varied greatly, ranging from 2.61% to 84.02% of all T-cells (median 54.6% of T-cells). In all four samples beyond day 74, no anti-BCMA^+^ T-cells were detectable. With Teclistamab interference, typically only a single T-cell population exhibiting increased mean fluorescence intensity in the anti-BCMA channel is observed (Fig. 1, lower right panel). In contrast, true positive samples following anti-BCMA CAR T-cell therapy display two clearly distinct T-cell populations (Supplemental Fig. S4, lower right panel).

**Fig. 2:**
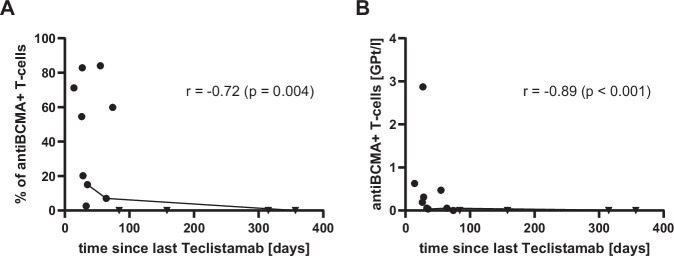
Time-dependent decline of anti-BCMA+ T-cells following Teclistmab cessation. Amount of anti-BCMA^+ a^ T-cells over time as percentage of all T-cells (**A**) and as concentration in GPt/l ^b^ (**B**). Dots represent measurements with detectable anti-BCMA^+^ T-cells, while triangles represent negative measurements. The three intra-patient measurements are connected by a line. A significant negative correlation between measurements and time since last Teclistamab application was observed, as indicated by the correlation coefficient r. Spearman's rank correlation was used as the data are not normally or log-normally distributed (Shapiro-Wilk test *p *< 0.01 for both subfigures). One-sided p-values are justified due to the biologically plausible decrease of anti-BCMA^+^ T-cells over time. ^a^BCMA: B-cell maturation antigen. ^b^ GPt/l: giga particles per liter (x10^9^/l).

Although the individual measurements varied widely, a negative correlation was detected between the amount of circulating anti-BCMA^+^ T-cells and the time since the last Teclistamab application, both in terms of the percentage of T-cells as well as concentration (Fig. 2). Notably, in the patient who provided three consecutive samples, a steady decrease in anti-BCMA^+^ T-cells was observed over time.

## Discussion

We demonstrated that Teclistamab impairs the detection of BCMA-CAR T-cells by assays based on labeled BCMA for at least 74 days (2.5 months). Serum-Teclistamab-concentrations show complex elimination kinetics of time-dependent and time-independent elimination with a decreasing Teclistamab clearance with ongoing therapy and high inter-individual differences based on body weight and myeloma characteristics [11]. This might explain the relatively weak negative correlation of anti-BCMA^+^ T-cells and time found in our study. It is also known that it can take up to 163 days for the Teclistamab concentration to decrease to 3% of C_max_ [11]. As far as we know, there are no data regarding the correlation of serum Teclistamab levels with Teclistamab binding on circulating T-cells. It can be assumed that Teclistamab binds to circulating T-cells with a high affinity and therefore is present on T-cells at least as long as it can be detected in the serum. We thus assume our findings of the disappearance of anti-BCMA^+^ T-cells after 2.5 months are in a reasonable range.

There are several approaches to circumvent the problem of anti-BCMA^+^ T-cells in the context of detecting CAR T-cells, including washing with Dithiothreitol (DTT), targeting other regions of the CAR construct by MFC, or using PCR-based methods. Due to the cytotoxic effects of DTT and the uncertain efficiency of complete Teclistamab displacement, we do not recommend using DTT when detecting rare events such as CAR T-cells. Depending on the CAR product used, alternative targets can be selected for MFC detection: All approved CAR products contain either a Whitlow or (G_4_S)_3_ linker, which can be detected by corresponding antibodies [12]. Also, the use of protein L [13] or polyclonal anti-F(ab) fragment [14] is possible. All of these methods have their limitations—for example, they may only work with specific CAR constructs or may also detect bispecific T-cell engagers that share the same linker structure.

We therefore recommend performing an MFC control measurement of anti-BCMA^+^ T-cells before BCMA-CAR application in all patients treated with Teclistamab in the last year. If negative, CAR-monitoring can be performed by MFC as usual. In case of false-positive anti-BCMA^+^ T-cells or uncertainty, a PCR-based method might be used to monitor CART-cells after recent Teclistamab cessation, even though availability is limited and costs are higher.

Most important, physicians and lab technicians should be aware of the problem of false-positive results in CAR T-cell detection assays and their growing relevance due to the increasing use of bispecific T-cell engagers. In theory, the same problem may occur after treatment with Elranatamab for anti-BCMA CARs or Blinatumomab for anti-CD19 CARs.

## Supplementary information

Supplemental Material

## Data Availability

The datasets and measurements analyzed during the current study are available from the corresponding author on reasonable request.
