# Supplementary material for: Teclistamab interference with anti-BCMA chimeric antigen receptor T-cell detection by flow cytometry: duration and clinical implications
Source: Leukemia. 2026 Apr 8;40(6):1327–30. doi: 10.1038/s41375-026-02961-y (PMC13233289; doi:10.1038/s41375-026-02961-y)
Supplement: Supplementary file 1 — Supplemental Material [file 41375_2026_2961_MOESM1_ESM.docx]

**Teclistamab interference with anti-BCMA Chimeric Antigen Receptor T-cell detection by flow cytometry: Duration and clinical implications**

Table S1 - Composition of antibodies used for multicolor flow cytometry

| target | fluorochrome | antibody | Tube 1  (FMO^a^ control) | Tube 2  (anti-BCMA-CAR^b^ detection) |
| --- | --- | --- | --- | --- |
| BCMA | none (biotinylated) | Milteny Biotec  Bergisch Gladbach,  Germany  Cat# 130-126-090 | none | 1 µl |
| CD45 | Horizon V500 | BD Biosciences  San Jose, CA, USA  Cat# 560777 | 5 µl | 5 µl |
| CD3 | Fluorescein isothiocyanate  (FITC) | BD Biosciences  San Jose, CA, USA  Cat# 345764 | 5 µl | 5 µl |
| Biotin | Phycoerythrin  (PE) | Milteny Biotec  Bergisch Gladbach,  Germany  Cat# 130-111-068 | 2 µl | 2 µl |

*^a^ FMO: fluorescence minus one
^b^ anti-BCMA-CAR: anti-B-cell maturation antigen chimeric antigen receptor*

| Sex | female = 3/13 (23.1 %)  male = 10/13 (76.9 %) |
| --- | --- |
| Age at sample collection | median 62.9 years  IQR 61.7 - 68.4 years |
| Body weight at last Teclistamab application | 81 kg  IQR 73 - 87 kg |
| Time difference to last Teclistamab application | median 55 days  IQR 28 - 84 days |
| Number of Teclistamab applications | median 18  IQR 8 - 24 |
| Cumulative dose of Teclistamab | median 2102 mg  IQR 1658 – 2885 mg |
| Last dosing regimen before cessation of Teclistamab | q1w 3/13 (23.1 %)  q2w 4/13 (30.8 %)  q3w 1/13 (7.7 %)  q4w 3/13 (23.1 %)  repeated step up 2/13 (15.4 %) |
| Peripheral blood leukocytes at sample collection | median 4.48 GPt/l  IQR 1.44 - 5.92 GPt/l |
| CD45^+^ events acquired in tube 1 | median 218 784  IQR 100 808 – 285 607 |
| CD45^+^ events acquired in tube 2 | median 255 214  IQR 214 341 – 379 701 |
| Percentage of anti-BCMA^+ a^ T- cells on all T-cells (tube 2 – tube 1) | median 15.05 %  IQR 0 - 59.87 % |

Table S2 - Overview of clinical data and sample properties

*^a^ anti-BCMA^+^: anti-B-cell maturation antigen positive*


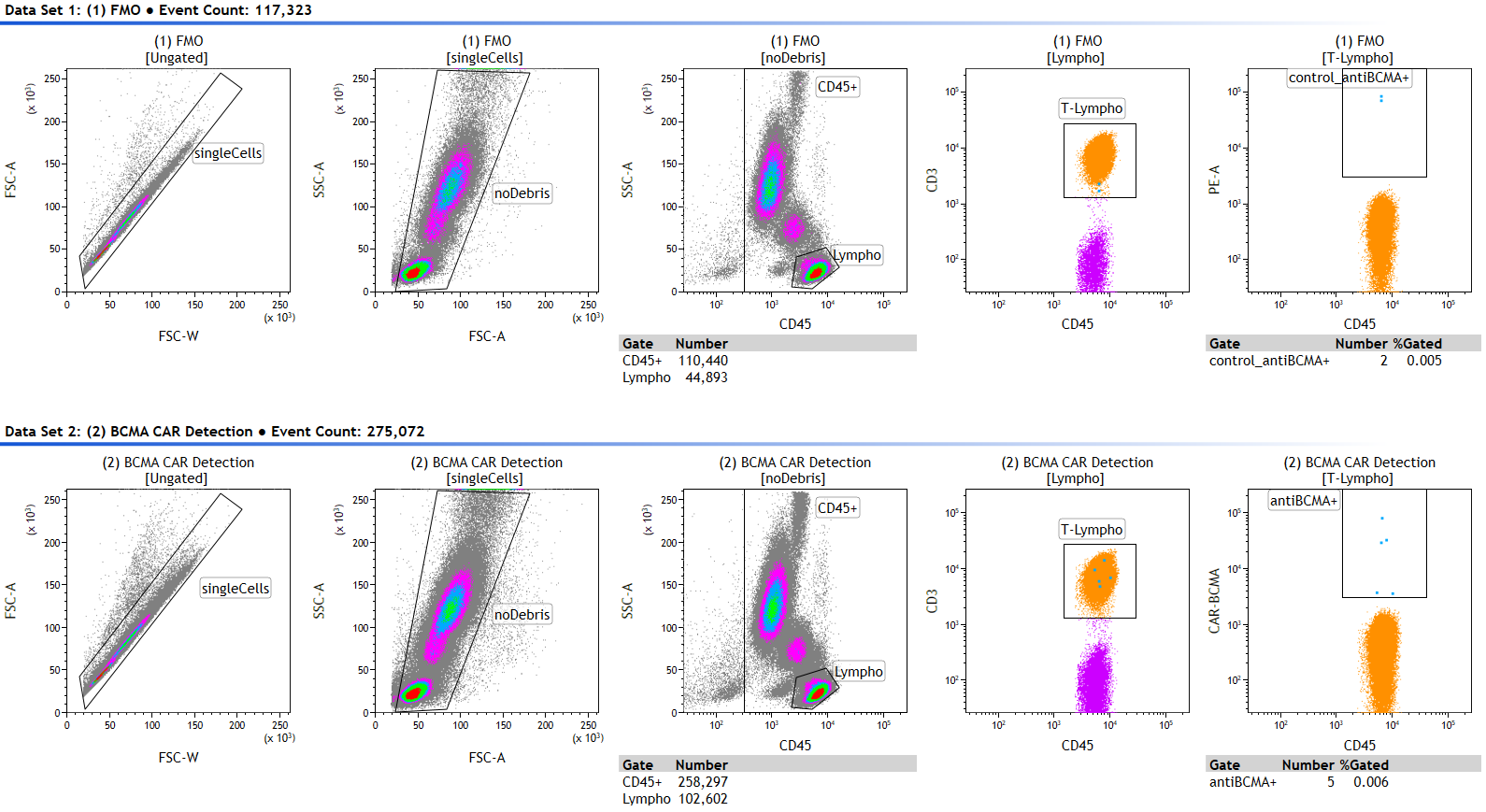


Figure S3 - Negative control example: anti-BCMA^+ a^ T-cells were not detected in a sample obtained from a healthy blood donor.

*^a^ anti-BCMA^+^: anti-B-cell maturation antigen positive*


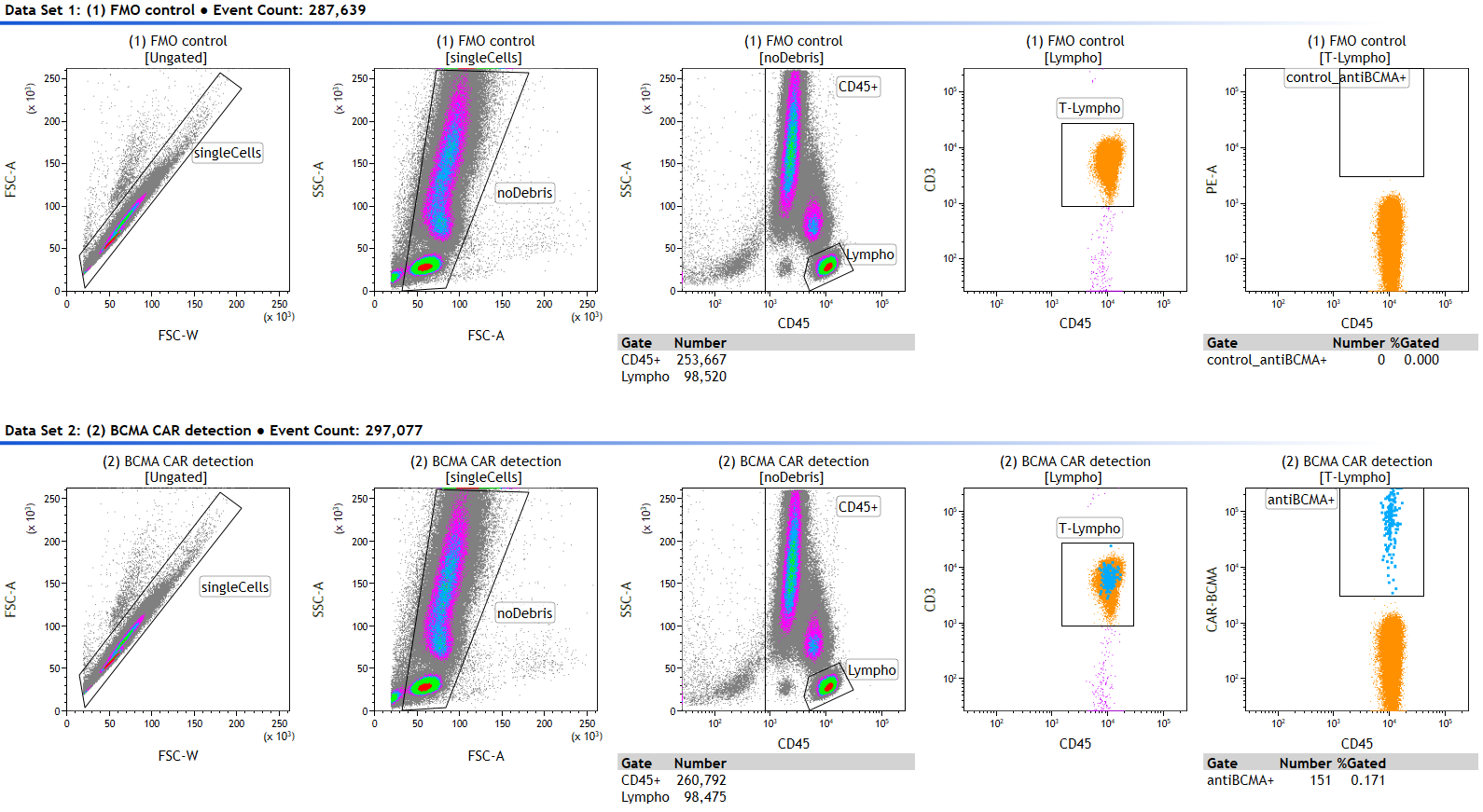


Figure S4 - Positive control example: anti-BCMA CAR^a^ T-cells are detectable in this patient at a frequency of 0.17 % of all T-cells, measured 386 days after treatment with Cilta-Cel. Two distinct populations can be seen in the lower right panel (true positive), whereas with Teclistamab interference (false positive), usually only a single population with increased mean fluorescence intensity is observed (see Figure 1 lower right panel).

*^a^ anti-BCMA CAR: anti-B-cell maturation antigen chimeric antigen receptor*
